# Long-term stable disease with mFOLFOX6 chemotherapy plus cetuximab for bone marrow metastasis from rectal cancer: A case report

**DOI:** 10.3389/fonc.2023.1117530

**Published:** 2023-01-26

**Authors:** Xiumei Fan, Fang Li, Chong Xiao, Yi Cai, Fengming You

**Affiliations:** ^1^ Oncology Department, Hospital of Chengdu University of Traditional Chinese Medicine, Chengdu, Sichuan, China; ^2^ TCM Regulating Metabolic Diseases Key Laboratory of Sichuan Province, Hospital of Chengdu University of Traditional Chinese Medicine, Chengdu, Sichuan, China

**Keywords:** bone marrow metastasis, rectal cancer, mFOLFOX6, cetuximab, long-term stable disease

## Abstract

Bone marrow metastasis from rectal cancer is a rare but severe disease associated with a poor prognosis due to limited treatment options. There is no consensus on therapeutic strategies, and better-tolerated and more effective treatment options are urgently needed. We report a case that one patient with rectal cancer developed pancytopenia 15 months after completion of radical surgery and chemotherapy and was diagnosed with bone marrow metastasis. The patient was treated with mFOLFOX6 chemotherapy plus cetuximab, considering both his poor bone marrow function and a genetic test showing a wild-type of KRAS/NRAS/PIK3CA/BRAF. Twelve cycles were successfully completed with dose modifications and supportive measures. The patient’s condition improved markedly based on a comprehensive assessment that included computed tomography images, blood cell counts, tumor markers, and clinical symptoms. The patient remains alive for 11 months at the last follow up. The patient treated with mFOLFOX6 chemotherapy plus cetuximab attained long-term stable disease, suggesting its promising efficacy and safety for bone marrow metastasis from rectal cancer and may hold promise as a treatment strategy for this specific patient population. Consideration can be given to the inclusion of mFOLFOX6 chemotherapy plus cetuximab in first-line treatment regimen for bone marrow metastasis from rectal cancer.

## Background

Colorectal cancer is the third most common cancer worldwide, and the trend of rectal cancer incidence increased (1). The liver and lungs are common sites of distant metastasis in rectal cancer, but rarely the cancer may metastasize to the bone marrow, which is a significant negative prognostic factor for survival (2). As bone marrow metastasis is easily confused with other blood-related diseases and is a risk for serious complications, such as bleeding and infections, accurate diagnosis and treatment of bone marrow metastasis arising from rectal cancer is challenging. Treatment options for patients with bone marrow metastasis from rectal cancer are limited and overall survival is shortened as a result (3). Currently, there is no standard of treatment, and it is imperative to develop effective therapeutic approaches for bone marrow metastasis from rectal cancer. Notably, combined chemotherapy and anti-EGFR therapy, such as cetuximab, can be attempted as a potentially life-prolonging therapy for bone marrow metastasis from rectal cancer (4).

Herein, we report the case of a 75-year-old patient with a bone marrow metastasis from rectal cancer who had previously undergone radical resection and postoperative adjuvant chemotherapy with XELOX. He was treated with mFOLFOX6 chemotherapy and cetuximab and showed durable long-term stable disease.

## Case presentation

A patient with rectal cancer underwent radical resection in March 2020 and five cycles of XELOX from June 2020 to September 2020. However, the patient failed to complete all eight cycles of chemotherapy because of severe gastrointestinal side effects.

The patient had low blood counts for about one month and presented to the hospital in January 2022. Blood cell counts indicated pancytopenia with a platelet count of 39 × 10^9^/L, a total hemoglobin level of 4.2 g/dL, a red blood cell count of 1.36 × 10^9^/L, and a white blood cell count of 3.45 × 10^9^/L. Meanwhile, there was a decline in the level of anti-internal factor antibody (0.97 AU/mL), serum vitamin B12 concentration (177 pg/mL), and serum folate concentrations (2.92 ng/mL). Furthermore, coagulation functions were abnormal with a fibrinogen level of 1.48 g/L, a thrombin time of 21.20 seconds, a D-dimer level of 15781 ng/mL, and an international normalized ratio of 1.40. Contrast-enhanced computed tomography (CT) showed no signs of metastasis. He was diagnosed with megaloblastic anemia, severe anemia, thrombocytopenia, and coagulation disorders at that time. Nevertheless, as he presented with an atypical clinical presentation, other diseases that can lead to low blood counts needed to be excluded, such as recurrent rectal cancer, paroxysmal nocturnal hemoglobinuria (PNH), Evans syndrome, leukemia, myelodysplastic syndromes, non-Hodgkin’s lymphoma, and myeloma.

**Figure 1:**
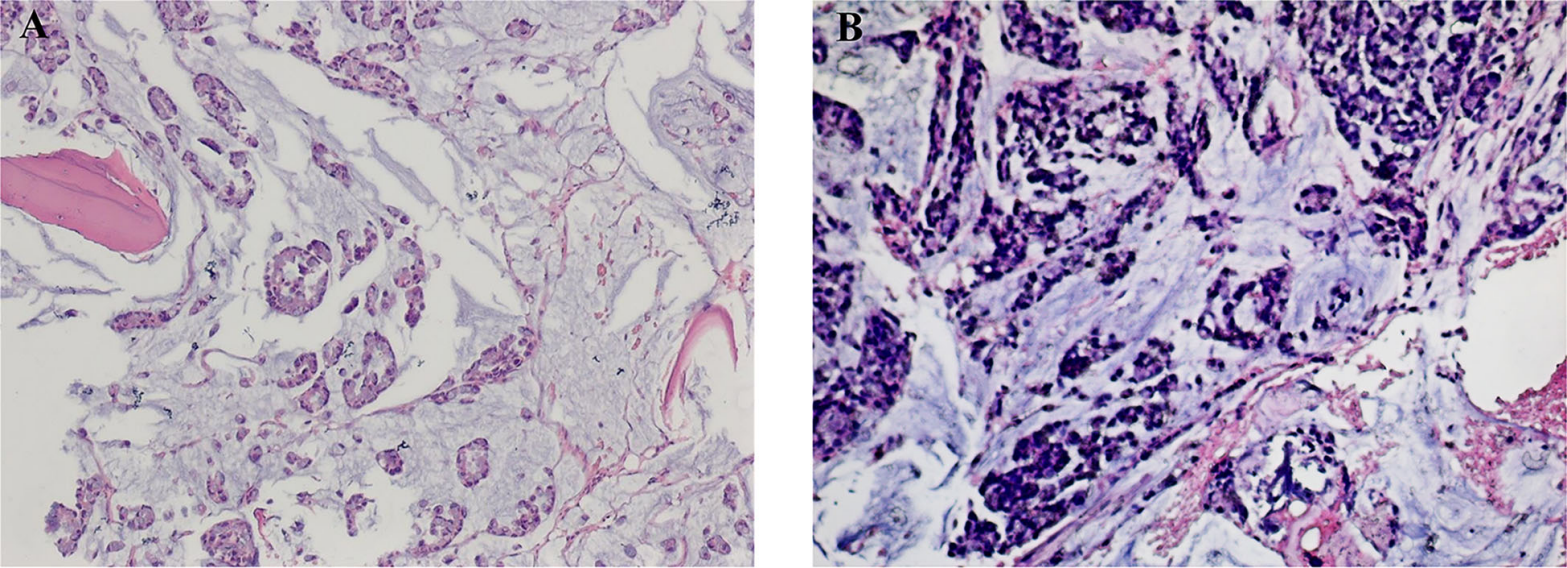
The results of a bone marrow biopsy pre- and post-treatment (H&E). **(A)** The pre-treatment bone marrow biopsy revealed metastatic adenocarcinoma with mucinous features. **(B)** The post-treatment bone marrow biopsy also shows metastatic adenocarcinoma.

Furthermore, specialty examinations for blood-related diseases were conducted. The patient was considered to have no PNH as neither CD55-deficient or CD59-deficient erythrocytes or granulocytes were present in his peripheral blood (as shown in **Supplementary Table 1**). There was no evidence from flow cytometry analysis of aberrant immunophenotypes associated with acute leukemia, high-risk myelodysplastic syndromes, non-Hodgkin’s lymphoma, or myeloma (as shown in **Supplementary Figure 1**). In addition, the BCR-ABL1 mutation was absent from all transcripts tested: p190, p210, and p230. Therefore, the patient was unlikely to have chronic myelogenous leukemia. Since both the direct and indirect Coombs tests were negative, Evans syndrome was ruled out. A bone marrow biopsy shows that a metastatic adenocarcinoma (**Figure 1A**). The results of immunohistochemistry staining were: CDX2 (+), villin (+), CK20 (+), CK7 (+), CK (-), PSA (-), HepPar1 (-), P63 (-), and TTF-1 (-) (as shown in **Supplementary Figure 2A**). Reticulin staining indicated that the bone marrow fibrosis was grade 1 (as shown in **Supplementary Figure 2B**). These results strongly suggested that the patient had bone marrow metastasis from rectal cancer. From this, it follows that severe pancytopenia originated from the invasion of the bone marrow by the primary tumor.

More specifically, a genetic test for rectal cancer was refined and tested for *KRAS*, *NRAS*, *PIK3CA*, and *BRAF*, which exhibited wild-type. The patient showed skin petechiae and asthenia, which were compatible with pancytopenia, and was treated with fresh frozen plasma and red blood cell transfusion prior to initiating chemotherapy. Laboratory work-up revealed a platelet count of 17 × 10^9^/L, a white blood cell count of 3.69 ×10^9^/L, a red blood cell count of 2.76 × 10^9^/L, and total hemoglobin levels of 8.5 g/dL after treatment, indicating improvement. While low blood cell counts may confer a high risk for further treatment, higher-risk therapies are needed to prolong survival. Considering both the genetic test results and the poor bone marrow function, the patient was treated with mFOLFOX6 chemotherapy plus cetuximab every 2 weeks. The patient received the first cycle of treatment in January 2022 and received prophylactic hemostasis at the same time, meaning that hemostats were given when the patient had thrombocytopenia but bleeding events did not occur. Twenty-four hours after completing the first cycle of treatment, the patient experienced bleeding from a stoma associated with thrombocytopenia and was treated with platelet transfusion and a Hemocoagulase Agkistrodon injection to stop the bleeding. In addition, the patient was transfused with red blood cells suspension, owing to an red blood cell count of 1.65 × 10^9^/L and a total hemoglobin level of 5 g/dL. Notably, when the patient was readmitted for the second cycle of chemotherapy, laboratory results showed a significant improvement in bone marrow hematopoiesis as indicated by a white blood cell count of 2.22 × 10^9^/L, an red blood cell count of 3.19 ×10^9^/L, a total hemoglobin level of 9.6 g/dL, and a platelet count of 108 × 10^9^/L. Nevertheless, due to chemotherapy-induced myelosuppression, the dose of the chemotherapy agent was reduced to 75% during the final three cycles of chemotherapy, while maintaining the initial planned dose of cetuximab. The patient showed excellent adherence and completed 12 treatment cycles. The patient’s treatment regimen is depicted in **Table 1**. In terms of adverse events, the patient experienced bleeding and chemotherapy-induced myelosuppression during treatment.

**Table 1:** A summary of blood cell counts and treatments.

Time	Blood cells				Transfusion support	Regimen adjustments	
	WBC (10^9^/L)	RBC (10^12^/L)	Hb (g/dL)	PLT (10^9^/L)			
11/01/22	3.45	1.36	4.20	39	N/A	N/A
12/01/22	N/A	N/A	N/A	N/A	fresh frozen plasma and red blood cell transfusion	N/A
17/01/22	3.69	2.76	8.50	17	N/A	N/A
18/01/22	N/A	N/A	N/A	N/A	hemostats	mFOLFOX6
22/01/22	N/A	N/A	N/A	N/A	platelet and red blood cell transfusion + hemostats	N/A
07/02/22	2.22	3.19	9.60	108	N/A	N/A
09/02/22	N/A	N/A	N/A	N/A	N/A	mFOLFOX6 + cetuximab
23/02/22	2.81	3.04	9.40	89	N/A	N/A
10/03/22	2.43	2.96	9.10	129	N/A	N/A
28/03/22	2.70	2.81	9	103	N/A	N/A
12/04/22	1.76	2.53	8	79	N/A	N/A
05/05/22	5.55	2.41	7.60	134	N/A	N/A
19/05/22	1.80	2.39	7.60	71	N/A	N/A
13/06/22	2.51	2.46	8	110	N/A	N/A
04/07/22	3.39	2.97	9.60	118	N/A	N/A
05/07/22	N/A	N/A	N/A	N/A	N/A	75% mFOLFOX6 + cetuximab
25/07/22	2.22	2.87	9.10	116	N/A	N/A
15/08/22	2.48	2.82	9	129	N/A	N/A

WBC, white blood cell; RBC, red blood cell; Hb, hemoglobin; PLT, platelet; N/A, not applicable.

Another bone marrow biopsy revealed persisting adenocarcinoma after the completion of therapy (**Figure 1B**). However, radiologic assessment using contrast-enhanced CT showed no metastases at other sites. Furthermore, after the completion of chemotherapy, there was a decrease in tumor markers, particularly in carcinoma antigen 125 and carcinoma antigen 199 (as shown in **Supplementary Figure 3**). Importantly, there was a marked improvement in hematopoiesis, and blood cell counts gradually returned to normal (**Table 1**). In addition, symptoms, such as loss of appetite, dizziness, fatigue, and asthenia, and the patient’s long-term quality of life were markedly improved by this treatment. The patient also consider that his condition was improved significantly after treatment. And the patient was still alive at the latest follow-up in November 2022. Currently, the patient has survived for more than eleven months after being diagnosed with bone marrow metastasis with the possibility of long-term survival.

## Discussion

Bone marrow metastasis from rectal cancer is an extremely rare disease that is challenging to diagnose and treat. Bone marrow metastasis is frequently associated with breast and gastric tumors but less often with rectal cancer (5). Diagnosing atypical bone marrow metastasis is difficult, and there is no consensus on standards for it. In this case, the patient manifested severe pancytopenia initially with no obvious predisposing factors. This condition can be easily confused with certain diseases in which patients can also present with reduced blood cell counts, such as PNH, Evans syndrome, leukemia, myelodysplastic syndromes, non-Hodgkin’s lymphoma, and myeloma. Consequently, it is important to maintain a broad differential diagnosis when the clinical features are not specific, and the diagnosis is uncertain. A few clinically important proteins are absent in PNH cells, such as CD55 and CD59 in granulocytes and erythrocytes, which can be used to differentiate between bone marrow metastasis from rectal cancer and PNH (6). For Evans syndrome, this autoimmune disorder is characterized by Coombs positivity and thrombocytopenia, which did not apply to this patient (7). In addition, leukemia, myelodysplastic syndromes, non-Hodgkin lymphoma, and myeloma can be identified using flow cytometry (8–11). Leukemias can be confirmed by genetic testing, such as the BCR-ABL fusion gene in chronic myelogenous leukemia (12). However, the radiologic findings of the patient were inconsistent with the pathology results since no radiological signs of bone marrow metastasis were present in our case, this result indicated that CT for bone marrow metastasis is not always sensitive and may lead to a missed diagnosis. Therefore, bone marrow biopsies should be performed to identify any bone marrow involvement in patient symptoms. A definitive diagnosis can provide firm support to determine suitable treatment options.

There are few reports that focus on the successful treatment of bone marrow metastasis from rectal cancer, and treatment selection remains controversial. Some clinicians have argued that chemotherapy should not be considered in patients with metastatic bone marrow disease because it may compromise already poor bone marrow function and not improve prognosis (4). However, achieving long-term survival is difficult in patients who do not receive chemotherapy, and there are those that believe chemotherapy should be considered. Patients with bone marrow metastasis from rectal cancer who received chemotherapy survived for longer than six months, but the vast majority of patients who did not died within three months (13). Additionally, the European Society of Medical Oncology consensus guidelines for colorectal cancer states that patients are eligible for chemotherapy despite their worsening condition due to bone marrow metastasis (14). Numerous studies have demonstrated that mFOLFOX6 efficacy in rectal cancer is well-established and that chemotherapy-mediated myelosuppressive effects are lower because of a lower dose of oxaliplatin (15). Importantly, Yoshida et al. found that mFOLFOX6 prolongs the survival of patients with bone marrow metastasis from rectal cancer (13). Nakamura et al. pointed out that anti-EGFR therapy with chemotherapy is beneficial for treating bone marrow metastasis from rectal cancer, even in patients with poor general status and prognosis (4). Moreover, cetuximab monotherapy has shown promising efficacy in patients with *KRAS*/*NRAS*/*BRAF* mutation-negative rectal cancer (16). Overall, mFOLFOX6 chemotherapy plus cetuximab can be used as a therapeutic strategy for patients with bone marrow metastasis from rectal cancer.

In our case, there were two challenging events that occurred during treatment. In the first cycle of treatment, prophylactic hemostasis was administered in conjunction with chemotherapy to avoid bleeding because of low platelet counts and clotting abnormalities. However, the patient experienced a hemorrhage, but hemostasis was successfully achieved by platelet and hemostatic agent transfusion. Second, due to poor bone marrow function and myelosuppression induced by chemotherapy, the blood cell counts decreased, and the chemotherapy dose was reduced by 25% during the final three cycles. Fortunately, by reducing the dose of the chemotherapeutic agent, there was a marked reduction in myelosuppression, and the last three cycles of treatment were successfully completed. No other adverse events were observed during treatment. After treatment, although a bone marrow biopsy revealed persisting adenocarcinoma, the patient’s condition improved markedly. Blood cell counts recovered to a relatively normal level, and there was a decline in tumor markers. In addition, the CT images did not show any tumor-associated abnormalities. More importantly, there was also a marked improvement in disease-related symptoms, such as dizziness, fatigue, and asthenia with an improved quality of life. The patient has survived beyond eleven months and remained alive. A comprehensive assessment of all results and clinical improvements suggests that the patient has long-term stable disease, and that the treatment regimen has a good efficacy and safety profile. FOLFORI was considered as a maintenance treatment for the patient after completion of treatment, but the patient declined further treatment due to poor economic status.

The patient prognosis improved after this combination treatment. A summary of the key findings of our case is presented here. First, if the etiology of a decreased blood cell count is unknown and conventional therapies are ineffective, early assessment of the bone marrow is imperative for more effective treatment and longer survival. Second, our case illustrates that the bone marrow cannot always be accurately assessed using CT; instead, biopsies can be used as sensitive, fast, and accurate tests. In addition, 18F-Fluorodeoxyglucose positron emission tomography/computed tomography also suitable for detecting bone marrow metastasis (4). In our case, further 18F-Fluorodeoxyglucose positron emission tomography/computed tomography was not conducted due to poor economic status. Third, although the patient with rectal cancer had a poor underlying condition due to bone marrow metastasis, receiving mFOLFOX6 chemotherapy and anti-EGFR therapy was necessary, valuable, and feasible. A key to the treatment strategy’s success is the timely adjustment of chemotherapy dosage based on bone marrow function. Fourth, as chemotherapy is administered when bone marrow function is poor, it is important to consider complications, such as thrombocytopenia-associated bleeding and leukopenia-associated infections, which should be aggressively prevented concurrently with chemotherapy initiation.

## Conclusion

Our case illustrates a case of long-term stable disease following mFOLFOX6 chemotherapy and cetuximab treatment in a patient with bone marrow metastasis from rectal cancer. Early diagnosis is the cornerstone for effectively treating this condition. Two critical factors are aggressive prevention and treatment of complications and timely adjustment of chemotherapy dosage based on bone marrow function. This treatment regimen showed promising efficacy and safety in our case, but there is a lack of high-quality, larger studies to validate these results. As a result, clinical and basic research is required to provide a well-established diagnosis and treatment protocols for bone marrow metastasis from rectal cancer.

## Data availability statement

The original contributions presented in the study are included in the article/**Supplementary Material**. Further inquiries can be directed to the corresponding authors.

## Ethics statement

This study was approved by the Medical Ethics Committee of Hospital of Chengdu University of Traditional Chinese Medicine. The patients/participants provided their written informed consent to participate in this study. Written informed consent was obtained from the individual(s) for the publication of any potentially identifiable images or data included in this article.

## Author contributions

XF conducted the study, provided patient care, collected clinical data, analyzed the data, and wrote the manuscript. FL conducted the study, supervised the research, collected clinical data, and wrote the manuscript. CX conducted the study, supervised the research, collected clinical data and analyzed the data. YC and FY conceived the study and reviewed the manuscript. All authors read and approved the final manuscript.
