# Supplementary material for: Long-term stable disease with mFOLFOX6 chemotherapy plus cetuximab for bone marrow metastasis from rectal cancer: A case report
Source: Front Oncol. 2023 Jan 26;13:1117530. doi: 10.3389/fonc.2023.1117530 (PMC9909532; doi:10.3389/fonc.2023.1117530)
Supplement: Supplementary file 1 [file DataSheet_1.docx]

**Supplementary Material**

**1 Supplementary Figures and Tables**

**1.1 Supplementary Figures**

**Supplementary** **Figure 1 Flow cytometry results of acute leukemia, high-risk myelodysplastic syndromes, non-Hodgkin’s lymphoma, and myeloma**

There was no immunophenotype abnormality of primitive or naive cells; CD34-positive cells made up approximately 0.35% of all nucleated cells with no immunophenotype abnormalities. The relative proportion of granulocytes was normal without abnormal expression of CD11b, CD13, CD15, or CD16. No abnormalities for monocytes was noted. There was a slight decrease in the proportion of lymphocytes but no immunophenotype abnormality in lymphocytes. T cells were 64.70% of lymphocytes, and the CD4/CD8 T cell ratio was 1.01. Mature B cells made up 7.11% of lymphocytes and were polyclonal B cells. There was no abnormal increase in the ratio of natural killer cells. There were 0.25% CD19-positive and CD10-positive naive B lymphocytes. No significant abnormalities in plasma cells were identified.

**Supplementary** **Figure 2 The results of CDX2 immunohistochemical and reticulin staining**

**(A)** Bone marrow revealing CDX2(+) indicated the metastatic adenocarcinoma was derived from the gastrointestinal tract. **(B)** Reticulin staining indicated that the bone marrow fibrosis was grade 1.

**Supplementary** **Figure 3 The changes in tumor marker levels during treatment**

There was a decrease in CA125 and CA199, indicating that the patient’s condition improved after treatment.

(Abbreviation: CA125, carcinoma antigen 125; CA199, carcinoma antigen 199)

**1.2 Supplementary Tables**

| **Supplementary** **Table 1 The** **expression of CD55/CD59 in erythrocytes and granulocytes** | | | |
| --- | --- | --- | --- |
| **Items** | **Detection method** | **Results (%)** | **Reference interval** |
| The ratio of CD55-negative erythrocytes to total erythrocytes | FCM | 0.62 | 0.00-5.00 |
| The ratio of CD59-negative erythrocytes to total erythrocytes | FCM | 0.42 | 0.00-5.00 |
| The ratio of CD55-negative granulocytes to total granulocytes | FCM | 0.65 | 0.00-5.00 |
| The ratio of CD59-negative granulocytes to total granulocytes | FCM | 0.52 | 0.00-5.00 |
| **Abbreviation:** FCM, flow cytometry | | | |
