# Supplementary figures and images for: Long-term stable disease with mFOLFOX6 chemotherapy plus cetuximab for bone marrow metastasis from rectal cancer: A case report
Source: Front Oncol. 2023 Jan 26;13:1117530. doi: 10.3389/fonc.2023.1117530 (PMC9909532; doi:10.3389/fonc.2023.1117530)

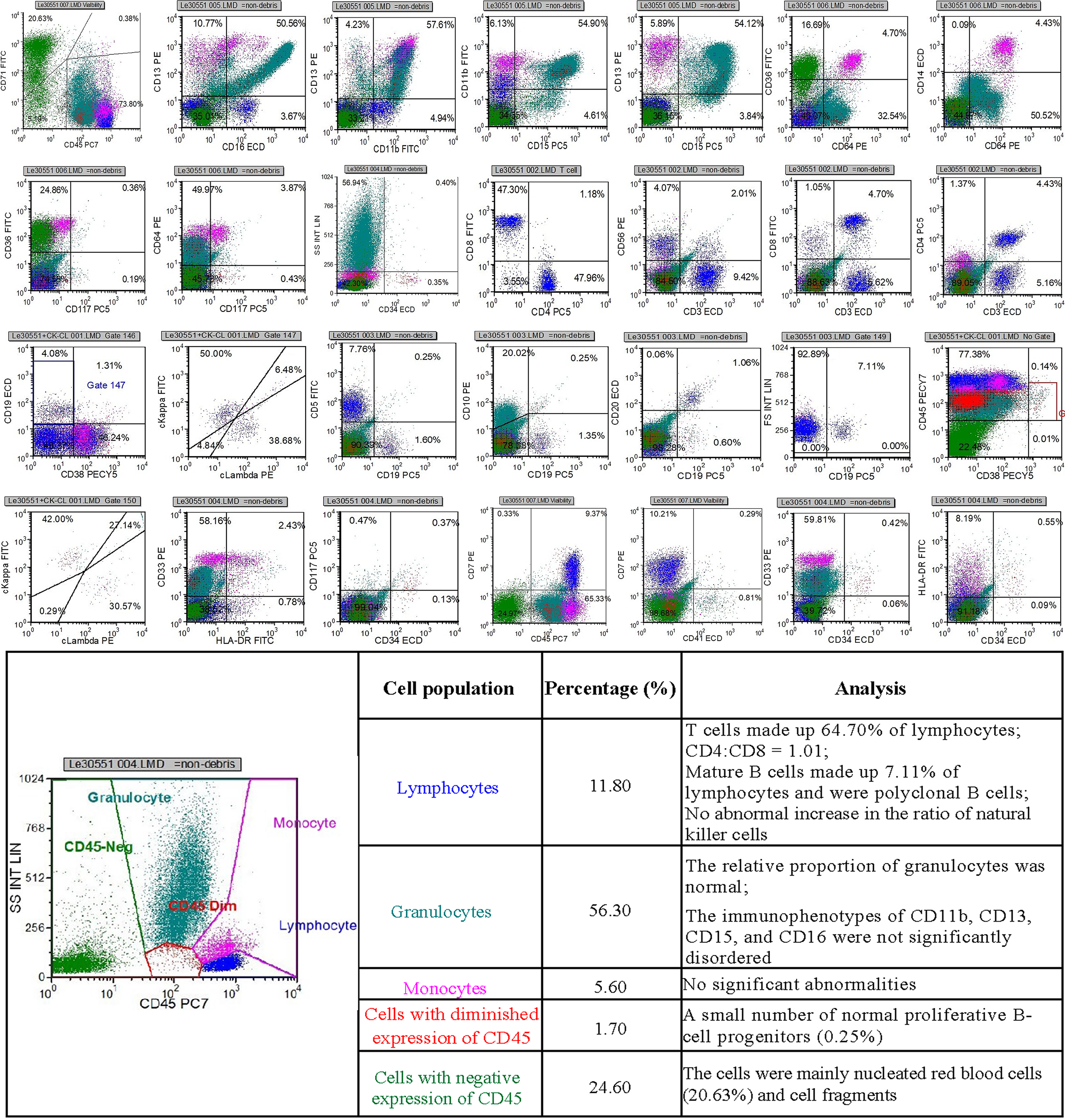

Supplement: Supplementary file 2 [file Image_1.tif]

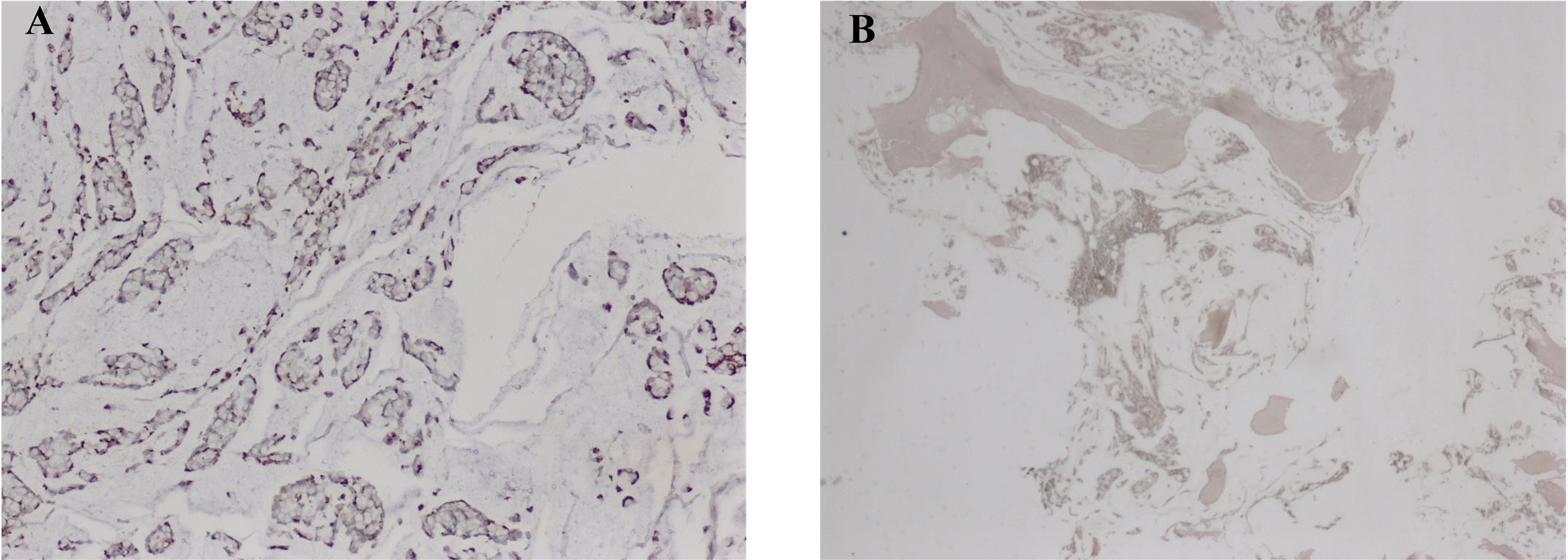

Supplement: Supplementary file 3 [file Image_2.tif]

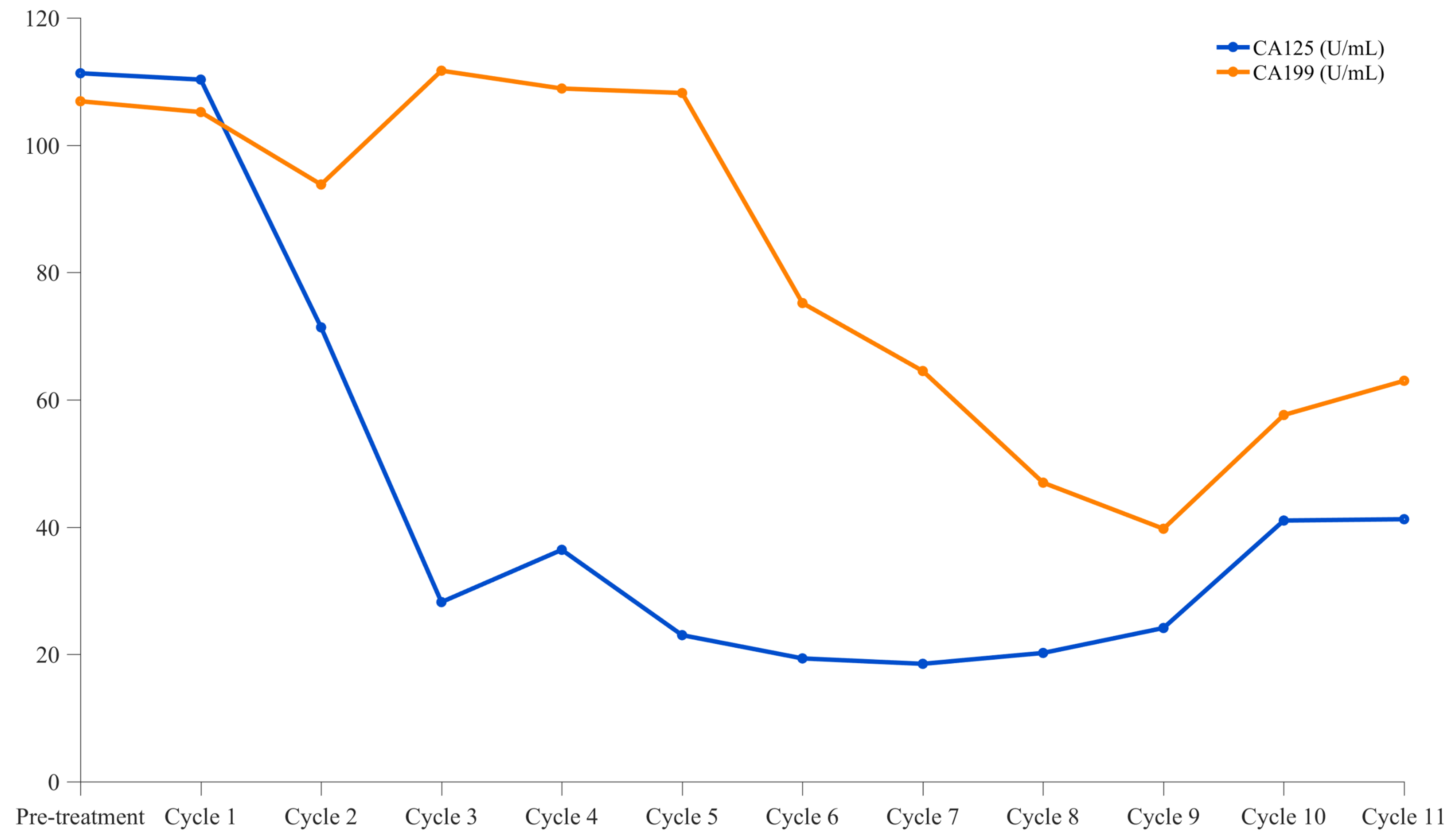

Supplement: Supplementary file 4 [file Image_3.tif]
